# Cardiorespiratory Fitness, Age, and Multiple Aspects of Executive Function Among Preadolescent Children

**DOI:** 10.3389/fpsyg.2020.01198

**Published:** 2020-06-10

**Authors:** Zhuxuan Zhan, Jingyi Ai, Feifei Ren, Lin Li, Chien-Heng Chu, Yu-Kai Chang

**Affiliations:** ^1^Key Laboratory of Adolescent Health Assessment and Exercise Intervention of Ministry of Education, East China Normal University, Shanghai, China; ^2^College of Physical Education and Health, East China Normal University, Shanghai, China; ^3^Department of Physical Education, National Taiwan Normal University, Taipei, Taiwan; ^4^Graduate Institute of Athletics and Coaching Science, National Taiwan Sport University, Taoyuan, Taiwan; ^5^Department of Physical Education, Beijing Language and Culture University, Beijing, China; ^6^Institute for Research Excellence in Learning Science, National Taiwan Normal University, Taipei, Taiwan

**Keywords:** executive function, working memory, inhibitory control, shifting, maturation, Progressive Aerobic Cardiovascular Endurance Run, cardiorespiratory fitness

## Abstract

Cardiorespiratory fitness (CRF) and age have been positively associated with children’s executive function; however, few studies have simultaneously assessed the associations between both variables and different aspects of executive function among preadolescent children. Therefore, the purpose of the current study was to evaluate the simultaneous influence of CRF and age on three aspects of executive function. Preadolescent children’s (*n* = 338) CRF levels were estimated based on the Progressive Aerobic Cardiovascular Endurance Run (PACER) test and then grouped into two age groups (Young Group: 9–10 years old and Old Group: 11–12 years old). Hierarchical multiple regression analyses were conducted for the 2-back task, the Flanker task, and the Local-Global task to assess the influence of CRF and age on working memory, inhibitory control, and shifting, respectively. Preadolescent children with greater CRF levels were associated with higher response accuracy during the 2-back task and shorter response time across congruent and incongruent conditions of the Flanker task, whereas older children showed generally superior cognitive performance. Notably, only the Old Group’s CRF was positively correlated with the accuracy in the switching condition of the Local-Global task. These findings suggest that CRF or age was generally associated with better performances in working memory and inhibitory control aspects of executive function. Furthermore, the positive influence of CRF on shifting may be modulated by developed cortical maturation.

## Introduction

Cardiorespiratory fitness (CRF), the direct indicator of individuals’ cardiovascular and respiratory systems’ overall capacity to perform physical activities, plays a critical role regarding physiological and psychological health. A higher CRF level has been linked to a lower metabolic syndrome risk and a cardiovascular disease risk (Twisk et al., 2000; Padilla-Moledo et al., 2012), increased volumes of certain cortical regions [e.g., the gray matter volume of the hippocampus and the basal ganglia (Chaddock et al., 2010a,b)], and lower risks of depression and anxiety (Biddle and Asare, 2011). The beneficial effects associated with higher CRF levels have been extended to academic performance in preadolescent school-aged children (Donnelly et al., 2016; Ruiz-Ariza et al., 2017; Chu et al., 2019). These improvements are possibly caused by CRF-related enhanced executive function, which is one of the foundations of academic performance (Hillman et al., 2009, 2014; Pontifex et al., 2011; Voss et al., 2011; Scudder et al., 2016; DiPietro et al., 2019).

Executive function is a top-down and meta-cognitive function required for conducting complex and goal-oriented operations. It consists of three distinct aspects: working memory, inhibitory control, and shifting (Miyake et al., 2000). Working memory, also known as updating, has been defined as the capacity of temporarily retaining relevant information and enables individuals to manipulate or further process this information (Miyake et al., 2000; Baddeley, 2012). Inhibitory control, also known as inhibition, refers to an individual’s ability to deliberately control, inhibit, or override a prepotent response or the ability to ignore irrelevant information or interferences in the environment and focus on relevant information. Shifting, also known as cognitive flexibility, represents the switching of attention or response strategies between mental sets according to the task demands (Miyake et al., 2000). These three aspects of executive function are highly important to preadolescent children’s learning and academic achievement (Kao et al., 2017a; Lippi et al., 2020).

A few studies have explored the associations between CRF and these three aspects of executive function separately (Tomporowski et al., 2015). Specifically, children with higher CRF assessed by the field-based CRF assessment [e.g., Progressive Aerobic Cardiovascular Endurance Run (PACER)] were associated with a superior performance on working memory capacity (Scudder et al., 2014, 2016). Cross-sectional research using the laboratory-based CRF assessment also revealed similar positive links between maximal oxygen consumption (VO_2max_) and working memory capacity (Kamijo et al., 2011; Drollette et al., 2016; Kao et al., 2017b). Similarly, cross-sectional studies utilizing either field-based (e.g., PACER) or laboratory-based (e.g., VO_2max_) CRF assessments to explore the associations between CRF and inhibitory control have revealed positive associations between CRF levels and task performance [e.g., shorter response times (Scudder et al., 2014; Drollette et al., 2016; Song et al., 2017) or higher response accuracy (Hillman et al., 2009; Pontifex et al., 2011; Scudder et al., 2014)], especially during the task condition requiring a more substantial amount of inhibitory control. Finally, similar findings of the beneficial effects of CRF on the shifting aspects of executive function have also been documented (Ishihara et al., 2018; Westfall et al., 2018).

Although relatively consistent evidence suggests a positive association between CRF and executive function, several potential limitations should be noted. First, prior studies examining the relationships between CRF and executive functions of children mainly used combined data across different age groups (e.g., aged 10–18 years) (Hillman et al., 2006; Themanson et al., 2006; Scudder et al., 2014; Kao et al., 2017b). Importantly, the cortical maturation trajectory of different parts of the brain are not uniform and, consequently, the development trajectory of various aspects of executive function might differ from each other during early childhood (Best and Miller, 2010; Diamond, 2013). It is plausible that the capability of completing the cognitive tasks increases while the structural and functional development continuously progresses throughout adolescence into early adulthood in which the associations between CRF and executive function might be altered in different age groups of preadolescent children. Due to the complex laboratory-based CRF assessment and neuro-electrical equipment, the sample sizes in prior research were relatively small and less representative (Scudder et al., 2014). Finally, relatively few studies have simultaneously compared the various aspects of executive function among preadolescent school-aged children.

Accordingly, the current research aims to examine how CRF and age influence the three aspects of executive function – working memory, inhibitory control, and shifting – in a large sample of preadolescent school-aged children. It was hypothesized that the children with higher levels of CRF or at older ages might be associated with superior executive function as reflected in better cognitive task performance, and then the interactions between CRF and age would be observed.

## Materials and Methods

### Participants

For this study, 377 school-aged preadolescent children were initially recruited. Eligible participants were screened by having to meet the following inclusion criteria: (1) age between 9 and 12 years old; (2) no history of psychiatric or neurological disease; (3) the anxiety scores assessed by the Chinese version of Self-Rating Anxiety Scale (Zung, 1971) were less than 50; (4) the depression scores assessed by the Chinese version of Children’s Depression Inventory (Yu and Li, 2000) were less than 19; (5) the scores of the modified Chinese version of Raven’s Standard Progressive Matrices were above the moderate levels; and (6) no history of cardiovascular disease.

Only those who completed the CRF and executive function assessments were included in the final analytical study. Three hundred thirty-eight children were finally recruited and grouped into the Young Group (9–10 years) and the Old Group (11–12 years) (Table 1).

**Table 1:** Preadolescents’ demographic and cognitive-related characteristics of the Young and the Old Groups (mean ± SD).

Variables	Young Group	Old Group
No. of participants (% male)	150 (62.7%)	188 (64.9%)
Mean age (years)	9.49 ± 0.50	11.37 ± 0.48
Height (m)	1.37 ± 0.07	1.48 ± 0.08^*^
Weight (kg)	30.07 ± 5.70	36.88 ± 7.99^*^
BMI (kg.m^−2^)	16.02 ± 2.10	16.73 ± 2.47^*^
Estimated VO_2max_ (ml.kg^−1^.min^−1^)	49.77 ± 3.48	46.71 ± 3.76^*^
Anxiety score	37 ± 6.36	42 ± 6.31
Depression score	12.35 ± 4.48	11.64 ± 4.58
Raven’s Standard Progressive Matrices score	53.7 ± 9.7	55.53 ± 3.63
2-back task
RT (ms)	1190.08 ± 242.9	1081.41 ± 230.09^*^
Accuracy	0.70 ± 0.17	0.79 ± 0.17^*^
Flanker task
Congruent RT (ms)	486.43 ± 108.71	463.37 ± 106.29
Congruent accuracy	0.69 ± 0.19	0.70 ± 0.19
Incongruent RT (ms)	503.47 ± 118.1	470. 67 ± 115.64^*^
Incongruent accuracy	0.65 ± 0.19	0.67 ± 0.19
Local-Global task
Non-switching RT (ms)	901.65 ± 293.26	836.14 ± 242.53^*^
Non-switching accuracy	0.90 ± 0.15	0.91 ± 0.14
Switching RT (ms)	1100.84 ± 332.42	1050.04 ± 275.63
Switching accuracy	0.86 ± 0.14	0.86 ± 0.14

VO_2max_, maximal oxygen consumption; RT, response time.

* *p* ≤ 0.05.

The study was approved by the Institutional Review Board of East China Normal University, China. The written informed consent was obtained from children and their legal guardians before the children participated in the experiment.

### CRF Assessment

In the current study, the PACER test, a multistage 20-m shuttle run test, was utilized to assess children’s CRF performance. The PACER test is considered valid for accurately measuring CRF for wide age ranges of participants, and it has been utilized in several prior studies that examined the association between CRF and cognitive and academic performance (Scudder et al., 2014, 2016). The test-retest reliability (*r* = 0.72–0.89) and validity (*r* = 0.51–0.84) of the PACER have been well documented previously (Leger et al., 1988; Mahoney, 1992; Yu and Fang, 2002).

The PACER test was administrated during participants’ second visits by the trained physical education teachers following the standardized protocol (Welk and Meredith, 2008). Briefly, participants were instructed to perform a 5-min standardized warm-up, following run back and forth from one maker to another marker spaced 20-m apart while keeping pace with the prerecorded cadence. The frequency of beat increases 0.5 km/h every minute, with an initial speed of 8.5 km/h. Participants were encouraged to keep up with the beat for as long as possible. The test was terminated if the given participant failed to traverse the 20-m distance between the markers within the designated time twice or was no longer able to maintain the pace and voluntarily stopped. The higher the number of laps a participant completed, the higher the CRF level they had.

Based on the number of the laps a child completed, the index of CRF (i.e., the estimated VO_2max_) was calculated using the following equation (Leger et al., 1988):

VO_2max_ (ml.kg^−1^.min^−1^) = 31.025 + 3.238 × speed (speed corresponding to the final stage in km.h^−1^) − 3.248 × age (years) + 0.1536 × age × speed

### 2-Back Task

The working memory aspect of executive function was assessed by the 2-back task that was programmed and ran using the E-prime software (v. 2.0, Psychology Software Tools, Inc.). The 2-back task is a modified *N*-back task, which has been a widely utilized paradigm to assess the capacity of working memory (Scudder et al., 2016; Ishihara et al., 2017). The 2-back task consisted of a sequence of white numeric digits (1–9), each measuring 1.5 cm × 1.5 cm. The stimulus was presented focally against a black background with a duration of 2,000 ms and a fixed intertrial interval of 3,000 ms. The task was composed of two blocks of 25 trials each, with 60 s of rest between the blocks. The total duration of the tasks summed to about 7 min. The participants were instructed to press the #1 key on the numeric keypad if the current stimulus (trial *n*) was matched to the stimulus two trials earlier (trial *n*−2); otherwise, they were instructed to press the #2 key. Trials of 50% probability as represented required the participants to press the #1 key as the correct response. The primary behavioral indices were the response times of the correct responses and response accuracy.

### Flanker Task

The inhibitory aspect of executive function was assessed by the modified Flanker task (Eriksen and Eriksen, 1974; Pindus et al., 2019) that was programmed and ran using the E-prime software. Each trial of the Flanker task consisted of a row of five parallel white arrows presented in the center of a 14″ LCD screen against a black background. The target arrow was posited in the middle, and two distractive arrows were on each side of the target arrow (flankers). Each trial appeared for 200 ms and then followed by a randomly selected various intertrial interval of 1,250–1,500 ms.

The Flanker task consisted of two types of trials: (1) the congruent trials (50%), in which the directions of all the arrows were the same (e.g., < < < < < or > > > > >) and (2) the incongruent trials (50%), in which the target arrow was pointed in the opposite direction from the flankers (e.g., < < > < < or > > < > >). The trials were separated into two blocks of 75 trials each, with 60 s of rest between the blocks. The overall number of congruent and incongruent trials were equal across the two blocks (50% of trials were congruent). The participants were instructed to press either the #1 or #2 key on the numeric keypad corresponding to the direction of the target arrow (left or right, respectively), as fast and accurately as possible.

If the participants responded within 200 ms, the stimuli disappeared, and the screen remained black for the rest of the 200 ms period. The response times of correct responses and response accuracy for congruent and incongruent conditions were recorded and assessed as the primary behavioral indices.

### Local-Global Task

The shifting aspect of executive function was assessed by the modified Local-Global task (Ishihara et al., 2018) that was programmed and ran using the E-prime software. The Navon-like global/local figures (Navon, 1977) were utilized during the task as the stimuli was made of two target numeric digits (i.e., the numeric digit 1, and 2) and two neutral distractors (i.e., the numeric digit 3 and 4). The large global numeric digits (global level) consist of copies of small local numeric digits (local level), and all of the numeric digits were presented equally at the global and local levels. For instance, 25% of the global numeric digits were the target numeric digit 1, which might be composed of one of the local distractor numeric digits. Similarly, 25% of the local numeric digits were the target numeric digit 1, which could be organized to form the global distractor numeric digits.

The switching condition (i.e., the target numeric digits were switched from local to global levels or vice versa) consisted of 46.8% of the trials. On the other hand, the repetitive non-switching condition (i.e., the current target numeric digit and the previous target were at the same level; e.g., a target “global” numeric digit 2 followed by a target “global” numeric digit 1) accounted for 53.2% of the trials. Two blocks of 36 trials each were presented, with 60 s of rest between the blocks. The stimuli presented focally on a 14″ LCD screen would disappear soon after the participants made their responses, and the next stimulus appeared immediately. Participants were instructed to press the #1 key or the #2 key on the numeric keypad when they identified the presence of target stimuli (the numeric digit 1 or the numeric digit 2, respectively) at either the global level or the local level. The stimuli remained on the screen until a response was made. The response time for a correct response and the accuracy of the switching and repetitive non-swathing conditions were the main behavioral indices.

### Procedures

Each eligible child participated for 3 separate days to complete the study. On their first visit, the children and their legal guardians completed the written informational concerns in a quiet indoor space. Additionally, their basic demographic data (e.g., gender, age, height, and weight) were collected or assessed. On their second visit, their CRF was assessed by the PACER test. On their third visit, three computer-based cognitive tasks were completed in a fixed order that lasted for an hour, with short breaks between cognitive tasks. The order of tests was as follows: the 2-back task, the Flanker task, and the Local-Global task. Before starting the formal cognitive tasks, the participants received detailed instructions regarding each cognitive task and have achieved at least 80% accuracy of the practice trials. Given the large number of children recruited for the current study, the primary researchers conducted the experiment during both morning and afternoon hours.

### Statistical Analysis of Data

Descriptive statistics were calculated to summarize the baseline characteristics of the participants. A series of independent *t*-test were further conducted by assessing the differences between the demographic and cognitive-related data by age group.

Separate 2-step linear hierarchical regression analyses were performed for two indices from the 2-back task (correct target response time/accuracy), and four indices from the Flanker task (correct congruent response time/accuracy and correct incongruent response time/accuracy), and four indices from the Local-Global task (correct switching response time/accuracy and correct non-switching response time/accuracy).

To examine the contributions of CRF, age, and their interactions on the three aspects of executive function, the variables were entered into the regression model in the following order: (1) CRF estimated from PACER laps, age group (coded as 0 for the Young Group and 1 for the Old Group), and body mass index (BMI) (Step 1) and (Step 2) the interactions between CRF and the age group (Step 2). If a significant interaction between CRF and an age group was detected, a simple slope analysis (Aiken et al., 1991) was performed to examine the main effects of CRF on the Young Group and the Old Group. The multiple regression coefficients squared *R*^2^ for the overall model (*R*^2^), the stepwise changes in *R*^2^ (Δ*R*^2^), and the standardized regression weight (*β*) for each predictor variable were reported. A significance level of *p* < 0.05 was set.

All statistical analyses were conducted using the SPSS® 21 statistical package (IBM Corporation, Armonk, NY, USA).

## Results

### Participants Characteristics

Independent *t*-test of the demographic data revealed that the participants in the Old Group were taller, heavier, and had larger BMI values than the participants in the Young Group (*p*s < 0.05). The Old Group also had lower estimated VO_2max_ values than the Young Group (*p* < 0.05). None of the other demographic variables were significantly different between the two groups (all *p*s > 0.05) (Table 1).

Independent *t*-test of the cognitive-related measures revealed that the Old Group had shorter response times and higher accuracy in the 2-back task, shorter response times in the incongruent Flanker task condition, and shorter response times in the non-switching Local-Global task condition than the Young Group (*p*s < 0.05). None of the other cognitive-related measures were significantly different between the two groups (all *p*s > 0.05).

### CRF, Age, and 2-Back Task

Regarding the response time, the regression analysis revealed a significant overall model effect of Step 1 (Δ*R*^2^ = 0.06, *p* < 0.01), with a significant negative effect for age (*β* = −0.25, *p* < 0.01) (Table 2). The overall model effect of Step 2 was not significant (*p* = 0.79); however, a significant negative effect for age (*β* = −0.26, *p* < 0.01) was observed.

**Table 2:** Regression analysis for the associations between cardiorespiratory fitness, age, the 2-back task, and the Flanker task.

	2-back task		Flanker task			
Model and variable			Congruent		Incongruent	
	Δ*R*^2^	*β*	Δ*R*^2^	*β*	Δ*R*^2^	*β*
Response time
Step 1	0.06^**^		0.03^**^		0.04^**^	
CRF		−0.06		−0.16^**^		−0.13^*^
Age		−0.25^**^		−0.16^**^		−0.17^**^
BMI		0.03		−0.08		−0.10
Step 2	<0.001		0.004		0.004	
CRF		−0.08		−0.08		−0.04
Age		−0.26^**^		−0.15^**^		−0.16^**^
BMI		0.04		−0.09		−0.11
CRF × Age		0.02		−0.10		−0.11
Accuracy
Step 1	0.07^**^		0.002		0.003	
CRF		0.13^*^		0.02		0.01
Age		0.29^**^		0.03		0.06
BMI		0.03		0.03		0.01
Step 2	0.01		0.002		0.001	
CRF		0.02		0.08		0.05
Age		0.28^**^		0.04		0.06
BMI		0.03		0.03		0.01
CRF × Age		0.13		−0.07		−0.05

BMI, body mass index; CRF, cardiorespiratory fitness.

* *p* ≤ 0.05;

** *p* ≤ 0.01.

Regarding the response accuracy, the overall model effect of Step 1 was significant (Δ*R*^2^ = 0.07, *p* < 0.01), with a significant positive effect for CRF (*β* = 0.13, *p* = 0.04) and for age (*β* = 0.29, *p* < 0.01). The overall model effect of Step 2 was not significant (*p* = 0.12); however, a significant positive effect for age (*β* = 0.28, *p* < 0.01) was observed.

### CRF, Age, and Flanker Task

Regarding the response time of the congruent condition, the overall model effect of Step 1 was significant (Δ*R*^2^ = 0.03, *p* < 0.01), with significant negative effects for CRF (*β* = −0.16, *p* < 0.01) and for age (*β* = −0.16, *p* < 0.01) (Table 2). The overall model effect of Step 2 was not significant (*p* = 0.23); however, a significant positive effect for age (*β* = −0.15, *p* = 0.01) was observed. Regarding the response accuracy of the congruent condition, neither the overall model effects of Step 1 and Step 2 nor other variables (CRF, age group, and BMI) were significant (all *p*s > 0.05).

Regarding the response time of the incongruent condition, the overall model effect of Step 1 was significant (Δ*R*^2^ = 0.04, *p* < 0.01), with significant negative effects for CRF (*β* = −0.13, *p* = 0.03) and age (*β* = −0.17, *p* < 0.01). The overall model effect of Step 2 was not significant (*p* = 0.22); however, a significant negative effect for age (*β* = −0.16, *p* = 0.01) was observed. Regarding the response accuracy of the incongruent condition, neither the overall model effects of Step 1 and Step 2 nor other variables (CRF, age group, and BMI) were significant (all *p*s > 0.05).

### CRF, Age, and Local-Global Task

Regarding the response time of the non-switching condition, the overall model effect of Step 1 was not significant (Δ*R*^2^ = 0.02, *p* = 0.07); however, a significant negative effect for age (*β* = −0.15, *p* < 0.01) was observed (Table 3). The overall model effect of Step 2 was not significant (*p* = 0.51); however, a significant negative effect for age (*β* = −0.16, *p* < 0.01) was observed. Regarding the response accuracy of the non-switching condition, neither the overall model effects of Step 1 and Step 2 nor the effects of the variables (CRF, age group, and BMI) were significant (all *p*s > 0.05).

**Table 3:** Regression analysis for the associations between cardiorespiratory fitness, age and Local-Global task.

Model and variable	Non-switching		Switching	
	Δ*R*^2^	*β*	Δ*R*^2^	*β*
Response time
Step 1	0.02		0.01	
CRF		−0.07		0.01
Age		−0.15^**^		−0.09
BMI		0.02		0.04
Step 2	0.001		0.003	
CRF		−0.12		0.07
Age		−0.16^**^		−0.08
BMI		0.02		0.04
CRF × Age		0.06		−0.09
Accuracy
Step 1	0.002		0.01	
CRF		0.01		0.08
Age		0.01		0.02
BMI		0.05		0.04
Step 2	0.003		0.01^*^	
CRF		−0.06		−0.07
Age		−0.001		0.01
BMI		0.05		0.05
CRF × Age		0.09		0.19^*^

BMI, body mass index; CRF, cardiorespiratory fitness.

* *p* ≤ 0.05;

** *p* ≤ 0.01.

**Figure 1:**
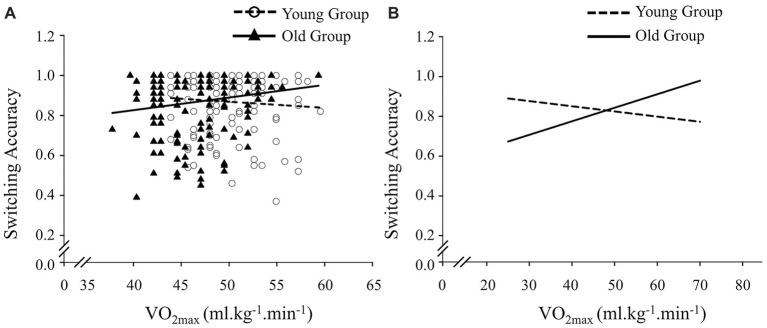
Scatterplots of the relationships between cardiorespiratory fitness (i.e., VO_2max_) and switching accuracy of the Local-Global task. **(A)** Raw data and **(B)** data that adjusted within the regression model.

Regarding the response time of the switching condition, neither the overall model effects of Step 1 and Step 2 nor the effects of the variables (CRF, age group, and BMI) were significant (all *p*s > 0.05). Regarding the response accuracy of the switching, neither the overall model effects of Step 1 nor the effects of the variables (CRF, age group, and BMI) were significant (all *p*s > 0.05). However, the overall model effect of Step 2 was significant (Δ*R*^2^ = 0.01, *p* = 0.03), with a significant positive effect for the interaction of CRF and age (*β* = 0.19, *p* = 0.03). Further decomposition of the interaction by the single slope analysis for each age group was carried out to test the significance of predicting the accuracy of CRF. The results revealed that CRF levels significantly associated with the higher switching accuracy among older children (*p* = 0.02), but the association was not observed in younger children (see Figure 1).

## Discussion

The current study was among the first large-sample investigations designed to better understand how CRF and age were associated with three aspects of executive function by using the 2-back task, the Flanker task, and the Local-Global task. The primary results revealed that CRF and age were positively associated with the performance of the 2-back task and the Flanker task. The interactions of CRF and age on the Local-Global task indicated the positive association between CRF and the performance of the switching condition in older preadolescents.

The findings of CRF being positively associated with higher accuracy in the 2-back task suggest that CRF is linked to working memory. Similar findings were also observed in CRF and working memory assessed by different tasks. Scudder et al. (2014) reported that young children who completed more laps were associated with superior working memory (response accuracy and working memory *d*’ scores) assessed using the field-based CRF (PACER test) and the spatial *N*-back paradigm. Laboratory-based assessed CRF (VO_2max_) has also revealed increased response accuracy and working memory *d*’ scores *via N*-back task (Drollette et al., 2016; Kao et al., 2017b). The subsequent 3-year longitudinal study further indicated that the positive correlations between increased CRF and enhanced working memory performance (the 2-back condition of the *N*-back task) were more potent at the more cognitively demanding task condition (Scudder et al., 2016). These studies that utilized different approaches to assess CRF and working memory provide a strong argument for the positive linkage between CRF and working memory from convergent perspectives.

The current study observed the association between CRF and inhibitory control aspect of executive function, regardless of the conditions of the Flanker task, being broadly consonant with prior research. In other words, children who completed a higher number of PACER laps were associated with better performance (i.e., shorter response times) in both congruent and incongruent conditions of the Flanker task relative to those completed few laps, suggesting a generally beneficial effect of CRF on inhibitory control among preadolescents (Scudder et al., 2014, 2016; Kao et al., 2017a; Raine et al., 2018; Westfall et al., 2018). Similar results showing an association between CRF levels and cognitive performance, regardless of the demands placed on executive function (including the differing demands of the congruent and incongruent conditions of the Flanker task), were also observed in elementary school children (Hillman et al., 2014; Scudder et al., 2014, 2016) and adolescents (Westfall et al., 2018). Collectively, the general improvements associated with CRF and inhibitory control-related tasks could be observed from preadolescent to adolescent children.

Along with CRF, our study also observed positive associations with age and working memory as well as age and inhibitory control, which are in line with prior developmental literature that the performance of working memory and inhibitory control continuously improved throughout childhood until the age of 15 years. The older children demonstrated superior working memory performance assessed by the *N*-back task and inhibitory control performance assessed by the Flanker task and evidenced by shorter response times or higher response accuracy (Best and Miller, 2010). Notably, a shorter response time without any of the negative impacts on the response accuracy for the Older Group suggests that age-induced increased performance does not result from the speed-accuracy trade off. In line with studies associated with CRF, these studies reflect a strong linkage between age and two types of executive function: working memory and inhibitory control. Our study has extended the existing knowledge by examining CRF and age simultaneously in preadolescent children.

The current findings have further demonstrated that age might moderate the correlation between CRF and shifting performance. Specifically, the positive relationship was only evident for older but not for younger preadolescent children during the more difficult parts of the task that require the upregulation of shifting ability. Our results have extended the previous research showing that 6–12-year-old children with a higher CRF were associated with shorter response times during the switching condition of the shifting paradigm (Ishihara et al., 2018) and have demonstrated that age might modulate the effects of CRF on shifting. The improvement of shifting ability (e.g., decreasing switching cost) usually continues into early adolescence following the inhibition and working memory processes (Huizinga et al., 2006; Best and Miller, 2010). A positive correlation between CRF levels in older adults and the performance of the task-switching paradigm (higher response accuracy for repeated and switching trials), which was mediated by the caudate nucleus volumes at the dorsal region (Verstynen et al., 2012), was observed. Thus, it is plausible that the limited beneficial effects of CRF on younger preadolescents were due to their lower levels of cortical maturation. Collectively, current findings have filled the gap of a lack of data concerning shifting and CRF in different ages of preadolescents (Verburgh et al., 2014) and have provided direct evidence of the performances on field tests of CRF and the shifting aspect of executive function.

To the best of our knowledge, this study was the first to investigate the CRF and working memory, inhibitory control, and shifting aspects of executive function simultaneously in a large sample of preadolescents from two age groups, thus making it possible to explore the associations between CRF, age, and more than one aspect of executive function in preadolescents. Despite the exciting findings from the current study, several limitations should be acknowledged. First, the CRF in the current study was assessed through the field-based PACER method. Although the maximum oxygen consumption (VO_2max_) has been considered as the gold standard for CRF assessment, the requirement of sophisticated equipment and the relatively high cost limited the application in the settings such as school and populational-based studies (Castro-Pinero et al., 2010). Second, the order of conducting the cognitive tasks was not counterbalanced across groups and individuals, and the fixed order might influence the results of current findings. Lastly, since the current study was cross-sectional, the directions of the associations between CRF levels, ages, and cognitive performance could not be determined. Future research would benefit from utilizing longitudinal observational or randomized control interventional approaches so that the influence of the changes in CRF on various aspects of executive function among preadolescent children over time could be elucidated, and the associated causality could be more clearly established.

In conclusion, children with higher levels of CRF or older children were generally associated with better working memory, inhibitory control, and shifting aspects of executive function. Notably, the interaction of CRF and age on shifting further suggests the role of developed cortical maturation in the relationship between CRF and executive function.

## Data Availability Statement

The datasets generated for this study are available on request to the corresponding author.

## Ethics Statement

The studies involving human participants were reviewed and approved by Institutional Review Board of East China Normal University, China. Written informed consent to participate in this study was provided by the participants’ legal guardian/next of kin.

## Author Contributions

Conceptualization: ZZ, C-HC, JA, FR, LL, and Y-KC. Methodology: LL, FR, and Y-KC. Formal analysis: ZZ, LL, and Y-KC. Investigation: ZZ, JA, and LL. Data curation: ZZ, FR, and JA. Writing – original draft preparation: ZZ, C-HC, JA, and Y-KC. Writing – review and editing: all authors. Visualization: ZZ and JA.

## Conflict of Interest

The authors declare that the research was conducted in the absence of any commercial or financial relationships that could be construed as a potential conflict of interest.
